# 3D‐printed scaffold composites for the stimuli‐induced local delivery of bioactive adjuncts

**DOI:** 10.1002/bab.2245

**Published:** 2021-09-06

**Authors:** Antonella Bandiera, Ovidio Catanzano, Paolo Bertoncin, Carlo Bergonzi, Ruggero Bettini, Lisa Elviri

**Affiliations:** ^1^ Department of Life Sciences University of Trieste Trieste Italy; ^2^ Department of Food and Drug Science University of Parma Parma Italy

**Keywords:** x

## Abstract

Polysaccharide scaffolds have been successfully employed to reconstruct environments that sustain skin tissue regeneration after injuries. Three‐dimensional (3D) advanced additive manufacturing technologies allow creating scaffolds with controlled and reproducible macro‐ and micro‐structure that improve the quality of the restored tissue to favor spontaneous repair. However, when persistent inflammation occurs, the physiological tissue healing capacity is reduced, like in the presence of pathologies like diabetes, vascular diseases, chronic infection, and others. In these circumstances, the bioavailability of therapeutic adjuncts like the growth factors in addition to the standard treatments represents undoubtedly a promising strategy to accelerate the healing of skin lesions. Precisely designed polysaccharide scaffolds obtained by 3D printing represent a robust platform that can be further implemented with the controlled delivery of bioactive adjuncts. Human elastin‐like polypeptides (HELPs) are stimuli‐responsive biopolymers. Their structure allows the integration of domains endowed with biological functionality, making them attractive compounds to prepare composites with smart properties. In the present study, 3D‐printed alginate and chitosan scaffolds were combined with the HELP components. The HELP biopolymer was fused to the epidermal growth factor (EGF) as the bioactive domain. Different constructs were prepared and the stimuli‐responsive behavior as well as the biological activity were evaluated, suggesting that these smart bioactive composites are suitable to realize multifunctional dressings that sustain the local release of therapeutic adjuncts.

**Abbreviations**
3D: three dimensional
AL: alginate
CH: chitosan
EGF: epidermal growth factor
HELP: human elastin‐like polypeptide
SDS‐PAGE: sodium dodecyl sulfate polyacrylamide gel electrophoresis

## 1 INTRODUCTION

Every organism has the intrinsic capacity to restore biological structures and functions after injuries. Skin tissue, being the protective barrier between the body and the environment, is the most exposed to damage and thus represents a paradigm for tissue regeneration.

Polysaccharide‐based hydrogels and scaffolds have been successfully employed to engineer the interface to provide biocompatible support promoting adequate healing conditions and tissue restoration. Chitosan (CH) and alginate (AL) among others are representative polysaccharides that exhibit high water‐retaining capacity, biodegradability, biocompatibility, and nontoxicity and have exhibited great potential for applications in skin tissue regeneration.^1^

The recent advance in three‐dimensional (3D) additive manufacturing technologies provides a major advantage to precisely define the inner geometries and structures of the polysaccharide scaffolds. These properties lead to the realization of new dressing with improved capacity to favor the healing of skin lesions.^2^, ^3^ However, depending on the level of tissue damage, systemic problems like metabolic dysfunction or other underlying pathologies may slow or even impair the healing process, leading to persistent or recurrent inflammation of the tissue.^4^, ^5^ In these circumstances, standard treatments often fail. On the other hand, tissue restoration relies on a multitude of growth factors and bioactive macromolecules, which are the essential regulatory mediators of the natural repair processes as the cutaneous skin repair.^6^, ^7^

Many studies have demonstrated the efficacy of adjuncts such as the growth factors when the normal physiologic process is impaired, like in nonhealing wounds.^8^, ^9^, ^10^ For this reason, these components have been employed to support conventional treatments for stimulating proper tissue repair.^11^, ^12^

For example, exogenous epidermal growth factor (EGF) was employed in skin tissue repair showing therapeutic efficacy and excellent tolerability.^8^, ^13^, ^14^ However, its topical administration is still challenging, due to local kinetic and pharmacodynamic limitations related to the harsh microenvironment in chronic wounds, which leads to enhanced degradation of exogenous growth factors.^15^, ^16^

However, the use of peptidic adjuncts, although representing a valuable therapeutic approach, is relatively limited at present. They are often not directly employable showing several drawbacks, including poor chemical and physical stability and a short circulating plasma half‐life.^17^ These aspects must be properly addressed for their use to treat conditions or diseases.

Multifunctional materials responding to environmental changes are attractive tools that provide valuable alternatives to standard therapeutic delivery. Options like the sustained and stimuli‐induced local release of bioactive agents represent some of the most promising ways to overcome the limits of the existing treatments.^18^, ^19^

The human elastin‐like polypeptides (HELPs)^20^ have been exploited to set up switchable systems suitable for controlling the delivery of substances of interest in the presence of elastolytic activity.^21^, ^22^ This is of special interest where persistent inflammation occurs, where the continuous neutrophils activation leads to an unfavorable microenvironment that impairs the spontaneous tissue repair as well as in other cutaneous conditions characterized by an altered elastolytic activity.^23^, ^24^, ^25^

Recent results pointed to the HELP polypeptides as particularly suitable compounds to obtain new composite materials that retain the main features of the original components.^26^ Moreover, HELP fusion products have been shown to maintain biological activity, and recently a fusion of this elastin‐like polypeptide with the EGF (then named HEGF) was described.^27^

The study reported here focuses on the development of new composites suitable for smart dressing fabrication that merge the flexibility of the 3D‐printing technique that allows the creation of finely tuned scaffolds by a layer‐by‐layer approach with the capacity of an elastolytic stimuli‐induced release of EGF of the HELP compounds. After the assessment of the interaction among 3D‐printed AL and CH scaffolds and HELP, the design and setup of the protocol to realize new composites was drawn up. Composites were further loaded with the bioactive HEGF and the elastolytic stimuli‐induced release of functional EGF was evaluated.

## 2 MATERIALS AND METHODS

### 2.1 3D‐printed AL and CH scaffolds

Sodium alginate (Ph.Eur. grade; molecular weight by gel filtration chromatography [GFC] 180–300 kDa; slowly soluble in water), acetic acid, and calcium chloride were from Carlo Erba (Milan, Italy). Chitosan (ChitoClear Fg90 TM1874 ‐ CAS 9012‐76‐4, degree of deacetylation 95%; molecular weight by gel permeation chromatography 150–200 kDa; allergen‐free, water‐insoluble, soluble in acid media) was from Primex (Primex EHF, Siglufjordur, Island).

Ink formulations for 3D printing were prepared as follows. Sodium alginate was suspended at the concentration of 6% (w/v) in a mixture of ultrapure water and 28% ammonia (95:5, v/v) to help polymer dissolution. CH powder was dispersed in acetic acid aqueous solution (2% v/v) at the concentration of 6% w/v. The suspensions were stirred magnetically for 24 h, then after complete dissolution of the polymer, 3D‐printed AL and CH scaffolds were prepared by the 3D‐printing technique, as already described.^28^ Briefly, a CAD (Computer‐Aided‐Design) model was designed employing SolidworksTM software (Dassault Systems, USA) by drawing a 3D grid consisting of overlapping parallel filaments set at a nominal distance of 200 μm. The whole object was composed of five layers of orthogonally disposed strands, having a theoretical total thickness of 1 mm. The.stl format file (STereo Lithography interface format) obtained was then processed and converted through Slic3rTM (RepRap) software in a.gcode format file, readable by the machinery.

The 3D‐printer surface plate was cooled at −14°C, while the viscous material (ranging 8000–40,000 cP) instantaneously solidified during construction layer‐by‐layer deposition.^29^ At the end of the printing procedure, the solid (frozen polymeric blend) structures were cross‐linked (CH: 30 min in 1.5 M KOH solution and AL: 30 min in 3 M CaCl_2_ solution, respectively) in their hydrogel form. Scaffolds were then washed with ultrapure water for the removal of cross‐linker excesses and stored at 4°C until use.

### 2.2 HELP and HEGF fusion protein production

The synthesis of HELPs was carried out as previously described.^20^ For the synthesis of HEGF, the synthetic gene of the HELP polypeptide was fused with the 53 aa coding sequence of the human EGF (GenBank AAS83395.1), exploiting the unique DraIII site in the expression vector that allows the in‐frame insertion at the C‐terminus of the polypeptide. The final construct was confirmed by sequencing. The recombinant products were expressed in the C3037 *E. coli* strain (New England Biolabs, Ipswich, MA). Expression and purification were carried out as already detailed.^27^ The recombinant products were analyzed by sodium dodecyl sulfate polyacrylamide gel electrophoresis (SDS‐PAGE) and the purified products were lyophilized for long‐term storage.

### 2.3 SDS‐PAGE and Western blot

Protein samples were dissolved in the loading buffer for few minutes and then loaded, without prior heating, on a 10% (w/v) reducing SDS‐PAGE that was run at 20 V/cm for 60 min. After electrophoresis, the protein bands were visualized by Coomassie Blue staining and photographed or transferred onto a PVDF membrane.

After blotting, the membrane was blocked using 5% (w/v) non‐fat dried milk in PBS solution containing 0.1% (v/v) Tween 20. Incubation was carried on in the same buffer with an anti‐human EGF polyclonal antibody (PeproTech) diluted 1:1000 for 16 h, and subsequently with secondary horseradish peroxidase‐conjugated antibody (Sigma‐Aldrich, #A6154) diluted 1:10,000 for 1 h. For His‐tag detection, a primary mAb (GE Healthcare, #274710‐01) was applied in the same conditions and it was recognized by a secondary anti‐mouse peroxidase‐conjugated antibody. Immunodetection was performed using the chemo‐luminescent substrate (ECLStar, EuroClone) according to the manufacturer's instructions.

### 2.4 HaCaT keratinocyte cells culture and metabolic assay

HaCaT keratinocyte cells were routinely grown in Dulbecco's modified Eagle's medium (DMEM) supplemented with 2 mM L‐glutamine, 100 μg/ml streptomycin, and 100 units/ml penicillin and containing 10% (v/v) heat‐inactivated fetal calf serum (FCS, Euroclone). Cells were maintained at 37°C in a saturated humidity atmosphere containing 5% CO_2_ in 25 cm^2^ flasks.

For estimating metabolic activity to evaluate the bioactivity of the samples of interest, cells were seeded at a density of 6000 cells/well in a 96‐well plate in 200 μl of DMEM complemented with 10% FCS. Twenty‐four hours after seeding, the medium was removed, washed with PBS, and substituted with 150 μl of fresh low FCS (0.2%) DMEM, in the absence (negative control) or the presence of the samples of interest, namely, HELP, HEGF, and the supernatants derived from the composite incubated with and without elastase. Human EGF (ReliaTech GmbH, Germany) was a positive control. Typically, a 1000× concentrated solution of the sample to assay was diluted with the low FCS medium to minimize possible aspecific effects of serum factors. The metabolic activity was evaluated at 72 h with the 2‐(4‐Iodophenyl)‐3‐(4‐nitrophenyl)‐5‐(2,4‐disulfophenyl)‐2H‐tetrazolium salt (WST‐1) provided in a pre‐mix electro‐coupling solution (Roche) according to the manufacturer's instructions. For this assay, 5 μl of the pre‐mix solution was added to each well. After 2 h, absorbance was read at 450 nm with a microplate reader (Synergy H1, BioTek, Winooski, VT).

### 2.5 Evaluation of 3D‐printed polysaccharide scaffolds interaction with HELP and HEGF

The capacity of CH and AL 3D‐printed scaffolds to adsorb proteins was evaluated by deposition of the HELP and HEGF polypeptides on the scaffolds.

Specimens were punched from random locations in the CH and AL 3D‐printed five‐layer sheets to obtain 5 mm‐diameter disks. These samples were frozen and lyophilized. When dry, 5 μl of a 3% (w/v) of either HELP or HEGF solution (150 μg of protein) was deposited on the disk and left for 1 h at ambient temperature in a wet chamber to avoid drying. Then, the samples were soaked in excess water (50 ml) for 2 or 16 h. Non‐washed controls were prepared in parallel. After each treatment, samples were incubated with 50 μl of 1× SDS‐PAGE loading buffer for 1 h at RT pulse‐vortexing every 15 min. Four microliters of these samples was used for SDS‐PAGE analysis.

### 2.6 Composites preparation

#### 2.6.1 3D‐printed polysaccharide scaffold inclusion in the HELP‐based matrix

The 5 mm‐diameter AL disk scaffolds were prepared as described in the previous subsection. Each scaffold disk was placed in the cap of a 0.5‐ml Eppendorf tube and a mixture of 20 μl of 4% (w/v) HELP in 10 mM Tris/HCl pH 8 solution plus 2 μl of 60 mg/ml of microbial transglutaminase solution was deposited on the wet scaffold. To obtain the EGF‐loaded composite, 1 μl of 4% (w/v) HEGF solution (corresponding to 40 μg) was added to the 20 μl of 4% (w/v) HELP solution. The tube was kept upside down and placed in an adaptor to be centrifuged for 3 min at 300*g*. The cross‐linking reaction was carried on for 2 h at RT and a further 16 h at 4°C in a wet chamber to avoid sample drying.

After cross‐linking, composites were washed with excess water, frozen, and lyophilized.

#### 2.6.2 HEGF loading in 3D‐printed AL scaffold

Four microliters of a 1% (w/v) HEGF water solution (corresponding to 40 μg) was deposited on a lyophilized 5 mm‐diameter AL disk scaffold. After quick incubation at RT (3 min), composites were washed with excess water, frozen, and lyophilized.

### 2.7 Scanning electron microscopy

The wet samples were frozen at −20°C and lyophilized. Slices were cut, mounted onto stubs using a double‐sided adhesive tape, and sputter‐coated with gold. Analysis was performed using a Leica Stereoscan 430i scanning electron microscope.

### 2.8 Evaluation of EGF release from HEGF‐loaded composites

The lyophilized composites described above were soaked in 500 μl of digestion buffer (50 mM Tris/HCl pH 7.5, 1 mM CaCl_2_) at 37°C for 16 h, the supernatant (named To/n) was sampled and stored at −20°C before analysis. The composites were then immersed in 700 μl of the 50 mM Tris/HCl pH 7.5, 1 mM CaCl_2_ buffer added of elastase from porcine pancreas (#E7885, Sigma‐Aldrich, ≥4 units/mg) to a final concentration of 0.5 μg/ml. Two hundred microliters of this supernatant was immediately removed (T0) and stored at −20°C for subsequent analysis. The composite was further incubated at 37°C in the remaining 500 μl with the elastase enzyme. After 2 h, 50 μl of supernatant was collected and stored at −20°C for subsequent analysis. For volume replacement, 50 μl of the fresh buffer with elastase was added to the sample to continue the incubation at 37°C. The same procedure was repeated after 4, 8, and 24 h.

The supernatants derived from the EGF release assays were analyzed by a dot blot procedure; 5 μl of the collected supernatants was spotted in four replicates onto a PVDF membrane that was previously soaked in methanol and washed in.

EGF (#SRP3027, Sigma Aldrich) solutions of 3 and 6 ng/μl corresponding to 15 and 30 ng/spot, respectively, were used as a calibration standard. The membrane was dried overnight at RT. After rehydration, the membrane was blocked for 1 h with 5% (w/v) non‐fat dry milk in 50 mM Tris/HCl pH 7.5, 0.5 M NaCl, 0.05% (v/v) Tween 20. The same buffer was used for primary antibody (polyclonal rabbit anti‐human EGF #500‐P45, PeproTech) dilution (1:1000). Subsequently, the secondary horseradish peroxidase‐conjugated antibody (Sigma) diluted 1:10,000 for 1 h was applied. After washing in 10 mM Tris/HCl pH 7.5, 0.15 M NaCl, 0.05% Tween 20, signals were developed by applying the chemoluminescent substrate (ECLStar, EuroClone). VWR Imager CHEMI Premium gel documentation and analysis system was used to capture and analyze images.

## 3 RESULTS AND DISCUSSION

### 3.1 Characterization of HEGF fusion protein bioactivity

**FIGURE 1:**
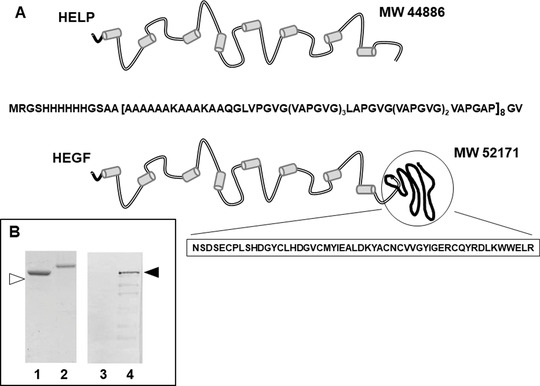
Stimuli‐responsive polypeptides. (A) Schematic representation of the primary structure of HELP and of the HELP‐based bioactive HEGF. (B) Immunochemical analysis of purified HELP (1 and 3) and HEGF (2 and 4) expression products. The two proteins were recognized with the anti‐His tag (1 and 2) and with the anti‐EGF antibody (3 and 4)

The HELP polypeptide was designed as a stimuli‐responsive fusion partner on which bioactive domains could be added by recombinant technology.^30^ In most cases, the elastin‐like final fusion protein presents the advantage of the purification by inverse phase transition maintaining the specific functionality of the added domain.^31^ The HEGF fusion protein has been recently employed to shed light on how immobilized active domains could influence the adhesion, survival, and differentiation of skeletal muscle cells.^27^ The schematic representation of the HELP and the HEGF recombinant proteins is shown in Figure 1A. In the HEGF protein, the human EGF sequence is fused with the HELP backbone. The final expression product is expected to maintain the elastin‐like properties as well as the susceptibility to degradation upon specific elastolytic stimuli and the EGF activity.

In the present study, we focused our attention on the realization of functional composite materials by combining the structure of the 3D‐printed polysaccharide scaffolds with the HEGF recombinant protein as the bioactive component, and very recent literature supports the advantages of this approach (Table 1).

**TABLE 1:** Impact of manufacturing techniques and new materials on controlled delivery of unconventional active principles

**Drugs and Growth factor delivery in wound healing**	Biomimetic nanoengineered scaffold for enhanced full‐thickness cutaneous wound healing. https://doi.org/10.1016/j.actbio.2021.01.029
	Recent advances in fiber‐hydrogel composites for wound healing and drug delivery systems. https://doi.org/10.3390/antibiotics10030248
	Emerging role of hydrogels in drug delivery systems, tissue engineering, and wound management. https://doi.org/10.3390/pharmaceutics13030357
	Epidermal and fibroblast growth factors incorporated polyvinyl alcohol electrospun nanofibers as biological dressing scaffold. https://doi.org/10.1038/s41598‐021‐85149‐x
	Externally triggered release of growth factors ‐ A tissue regeneration approach. https://doi.org/10.1016/j.jconrel.2021.02.015
	Spatially heterogeneous epidermal growth factor release from microporous annealed particle (MAP) hydrogel for improved wound closure. https://doi.org/10.1039/d1tb00715g
	Diving into 3D (bio)printing: A revolutionary tool to customize the production of drug and cell‐based systems for skin delivery. https://doi.org/10.1016/j.ijpharm.2021.120794
**Manufacturing for drug delivery**	Review on computer‐aided design and manufacturing of drug delivery scaffolds for cell guidance and tissue regeneration. https://doi.org/10.3389/fbioe.2021.682133
	Three ‘D's: design approach, dimensional printing, and drug delivery systems as promising tools in healthcare applications. https://doi.org/10.1016/j.drudis.2021.06.016
	Polymer 3D printing review: materials, process, and design strategies for medical applications. https://doi.org/10.3390/polym13091499
	Advanced strategies for tissue engineering in regenerative medicine: a biofabrication and biopolymer perspective. https://doi.org/10.3390/molecules26092518
**Smart stimuli responsive systems**	Bioresorbable polymers: advanced materials and 4D printing for tissue engineering. https://doi.org/10.3390/polym13040563
	Medical application of biomimetic 4D printing. https://doi.org/10.1080/03639045.2020.1862179
	Externally triggered release of growth factors ‐ A tissue regeneration approach. https://doi.org/10.1016/j.jconrel.2021.02.015
	Review of applications and future prospects of stimuli‐responsive hydrogel based on thermo‐responsive biopolymers in drug delivery systems. https://doi.org/10.3390/polym13132086
**Elastin‐inspired materials**	Elastin‐inspired supramolecular hydrogels: a multifaceted extracellular matrix protein in biomedical engineering. https://doi.org/10.1039/d0sm02202k
	Fibrous scaffolds from elastin‐based materials. https://doi.org/10.3389/fbioe.2021.652384

Due to the presence of the HELP moiety, the resulting material is expected to degrade upon specific proteolytic stimuli releasing the bioactive EGF factor. The EGF domain corresponds to approximately one‐tenth of the HEGF macromolecule. In Figure 1B, the immunochemical analysis showed that both HELP and HEGF recombinant products were recognized by the anti‐His tag antibody, whereas only HEGF was recognized by the anti‐EGF antibody, confirming the presence of the EGF domain in this expression product. Due to the sensitivity of this last antibody, a slight degradation pattern is barely observable in this sample, related to its repetitive feature, as already described for other elastin‐like polypeptides.^32^

**FIGURE 2:**
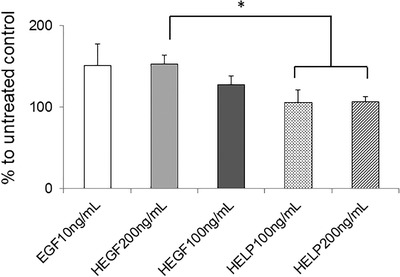
Biological activity of the HELP‐based polypeptides. HaCaT cell cultures were treated with the indicated amount of each expression product. Metabolic activity was evaluated at 72 h by 2‐(4‐iodophenyl)‐3‐(4‐nitrophenyl)‐5‐(2,4‐disulfophenyl)‐2H‐tetrazolium salt (WST‐1) assay. Untreated and EGF‐treated cells were the negative and the positive control, respectively. Results are expressed as mean ± standard deviation; the analyses were performed at least in quadruplicate. *p < 0.05

The biological activity of the HEGF protein was assessed. The effects of HEGF on metabolic activity and proliferation of cells were already evidenced on C2C12 myoblasts.^27^ Because this work aimed to realize composite materials that can favor the repair of hard‐to‐heal skin lesions, we performed the study on the HaCaT keratinocyte cell line, a well‐recognized in vitro model that is commonly used to assay the response of human keratinocytes.^33^ First, the proliferative effect of the whole HEGF fusion protein added to the cell culture medium was compared to that of human EGF as a reference or to that of HELP alone. In Figure 2, HEGF showed a dose‐dependent effect comparable to that of EGF on the proliferation of HaCaT cells, whereas HELP alone showed only a slight effect that was not dose‐dependent.^27^, ^34^ These results confirmed that the expressed HEGF fusion maintained the biological function of the EGF domain toward the HaCaT cells. The HEGF protein showed less activity than EGF, however, in addition to the difference of size of the two polypeptides, it is not surprising that the presence of the HELP backbone could affect the activity of the bioactive EGF domain. However, these data showed that the HEGF fusion represents a relevant starting material for the design and fabrication of composites endowed with specific bioactivity.

### 3.2 Evaluation of interaction between 3D‐printed polysaccharide scaffolds and HELP polypeptides

**FIGURE 3:**
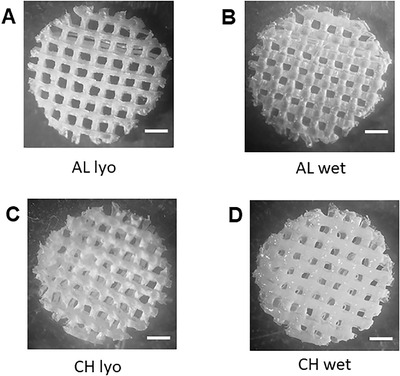
3D‐printed polysaccharide scaffolds used in this study. Scaffolds were composed of alginate (A and B) and of chitosan (C and D) as detailed in Materials and Methods section. The scaffolds maintained their structure after lyophilization (A and C) and after subsequent rehydration (B and D)

**FIGURE 4:**
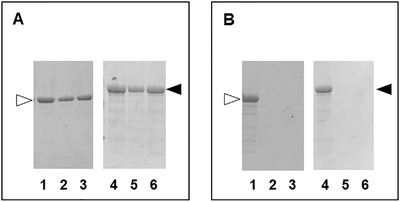
Representative image of electrophoretic analysis of the 3D‐printed polysaccharide scaffolds loaded with the HELP‐based polypeptides. Alginate (A) or chitosan (B) scaffolds were loaded with 150 μg of HELP (lanes 1–3, white arrow) or HEGF (lanes 4–6, black arrow). Protein content was extracted before (lanes 1 and 4) or after quick (lanes 2 and 5) and prolonged (lanes 3 and 6) water wash. The protein extracts were analyzed by SDS‐PAGE, Coomassie blue staining

The 3D‐printing process, allowing the control of the structural architecture, holds great potential in producing tunable polysaccharide scaffolds for biomedical applications.^28^ It was previously observed that 3D‐printed polysaccharide scaffolds have shown improvement in terms of biological performance compared with the standard commercial patches.^35^ The 3D‐printed scaffolds represent a good starting point to realize bioactive composites with HELP‐based matrices. The 3D‐printed CH and AL scaffolds were selected to study the interaction and compatibility with HELP‐based macromolecules. These scaffolds maintained their multilayer 3D‐printed structure after the lyophilization procedure and subsequent re‐wetting (Figure 3). This feature was functional to load the HELP macromolecules within these supports. Lyophilized scaffolds of CH or AL were thus re‐hydrated with an aqueous solution containing the HELP polypeptide. The same procedure was employed to load scaffolds with the HEGF. Control replicas were let to dry, whereas the still wet samples were extensively washed. Proteins were extracted both from controls and from the washed samples and were analyzed by SDS‐PAGE. The results are shown in Figure 4. When loaded with each protein, the 3D AL scaffolds retained the most of HELP and HEGF even after a prolonged wash (Figure 4A). On the contrary, both proteins were removed even after a 2‐h wash from the CH scaffold (Figure 4B).

These results showed that both scaffolds could be loaded with HELP‐based proteins. However, the remarkably different behavior of the scaffolds toward the proteins suggested a different interaction related to the nature of the component of the scaffold itself. Likely, the carboxylic acid groups that confer negative charges to AL can interact electrostatically with the positively charged cross‐linking domains of the HELP moiety, whereas the CH chains, bringing the same charge, hinder the interaction with HELP.

### 3.3 Design and preparation of 3D‐printed polysaccharide scaffold‐based composites

**FIGURE 5:**
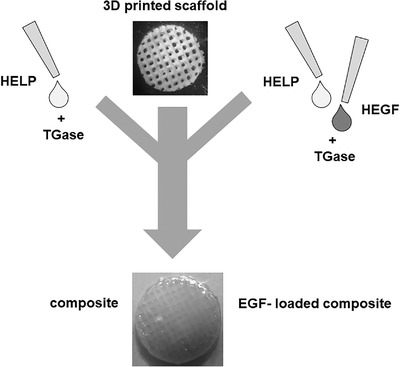
Schematic representation of the setup for preparation of composites composed of 3D‐printed polysaccharide scaffold and HELP‐based matrices

**FIGURE 6:**
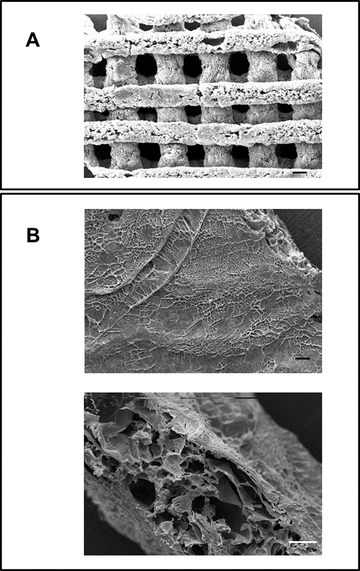
Scanning electron microscopy analysis of composites including 3D‐printed alginate (AL) scaffold and HELP‐based matrices. Representative SEM images of AL scaffold alone (A) and AL/HELP matrix composite (B). The appearance of the surface of the composite at the same magnification as in (A) is shown in (B), upper panel. The section of the composite disc is shown in B, lower panel. Bar is 100 μm

A method for preparing composites made up of 3D polysaccharide scaffolds and HELP biopolymers was set up, based on the preparation of the HELP hydrogel matrix.^36^ The procedure is schematically shown in Figure 5. The scaffolds, either of AL or CH, were both successfully embedded in the HELP‐based hydrogel employing the transglutaminase enzyme,^36^ as detailed in Materials and Methods. The macroscopic appearance of these composites is shown in Figure 5. Details of the microscopic structure observed by SEM are shown in Figure 6. The whole 3D structure of the printed scaffold (Figure 6A) was successfully embedded in the HELP matrix as can be observed in Figure 6B (upper panel). The structure of the 3D‐printed scaffold is covered by a layer of hydrogel matrix. The HELP matrix penetrated all the space between the 3D‐printed scaffold grids filling the void spaces of the scaffold, resulting in a continuous microporous structure (Figure 6B, lower panel). All these constructs could be soaked in water overnight without observable change. Notably, this route of preparation is suitable for integrating the bioactive HEGF polypeptide in the composite just adding it to the HELP solution that undergoes the enzymatic cross‐linking, as schematized in Figure 5.

Although both AL and CH scaffolds were suitable to prepare this kind of composite, the AL scaffold offered two ways to incorporate the HEGF within the constructs. As described in the previous paragraph, the HEGF was integrated with this scaffold both exploiting the tight HELP/AL electrostatic interaction (Figure 4A) and by the enzymatic cross‐linking within the HELP matrix (Figure 5), allowing the realization of stimuli‐responsive composites endowed with bioactive potential.

### 3.4 Stimuli‐induced release of bioactive EGF from composites

**FIGURE 7:**
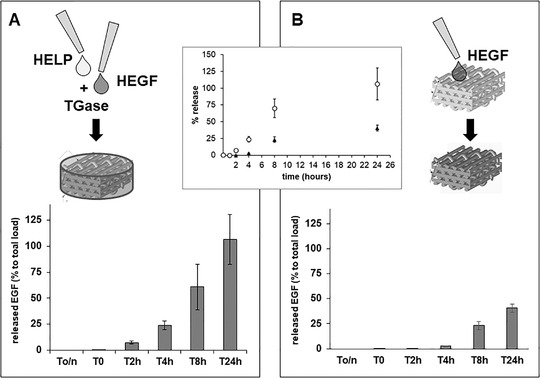
Cumulative release of EGF from the alginate (AL) composites. Two AL composites were tested for EGF release in the presence of elastolytic activity. HEGF was added to the matrix‐forming mixture of HELP and transglutaminase that embedded the scaffold (A) or it was directly dropped on the lyophilized scaffold (B). The release kinetics were assessed by immunodot blot as detailed in the text. To/n is referred to the supernatant sampled as detailed in Materials and Methods. In the inset, the kinetic of the release of the composite schematized in A (open circles) and of the composite schematized in B (black circles) is evidenced. Results are expressed as mean ± standard deviation and are representative of at least three independent experiments

The composites based on the HELP matrix described above have the intrinsic property to respond to environmental changes, in particular by sensing the presence of elastolytic activity.^21^, ^22^ In this condition, the composites are expected to undergo degradation, releasing the bioactive domains. To assay the capacity of the HELP‐based composites to release the bioactive fraction, that is, the functional EGF domain, we focused our attention on the constructs prepared with the AL scaffold. Thus, two series of bioactive composite specimens were prepared, one in which the bioactive component HEGF was integrated into the scaffold‐embedding HELP matrix (Figure 7A) and the second one in which the HEGF was adsorbed on the scaffold (Figure 7B).

First, the constructs were washed with excess water to ensure the removal of any unbound or unreacted component. Then, to assess the capacity of EGF release, the constructs were soaked for 16 h at 37°C (To/n) in the buffer alone, before the incubation in the presence of the enzyme that triggers the release (see details in Materials and Methods). At different times, the supernatants were analyzed by the immune‐dot blot recognition to detect the EGF that was released in the medium. As shown in Figure 7A,B, the supernatant derived from the 16‐h incubation of the constructs in the absence of the elastase (To/n) did not show any signal. On the contrary, after enzyme addition, signals became detectable after 2 or 4 h of incubation, depending on the type of composite. Interestingly, the release of EGF showed a striking difference between the two HEGF‐loaded AL scaffold composites. After 24 h, 100% release of the loaded EGF from the HELP/HEGF matrix‐embedded constructs was achieved (Figure 7A), whereas about 50% was released from the other type of construct (Figure 7B) at the same time. Moreover, the release kinetic from the HELP/HEGF matrix‐embedded constructs (Figure 7A) resulted significantly faster (almost one and a half time) than that of the second type of construct, on which HEGF was directly adsorbed (Figure 7B). Likely, in these last constructs, the action of the elastase enzyme present in the medium was hampered by the presence of the AL scaffold fibers, resulting in its slowdown. On the contrary, the enzymatic action on the HELP/HEGF matrix‐embedded construct could promptly start, likely due to the ease of access to its target.^21^

After the assessment of the presence of EGF in the medium, the functionality of the products of the elastase degradationwas assayed.

**FIGURE 8:**
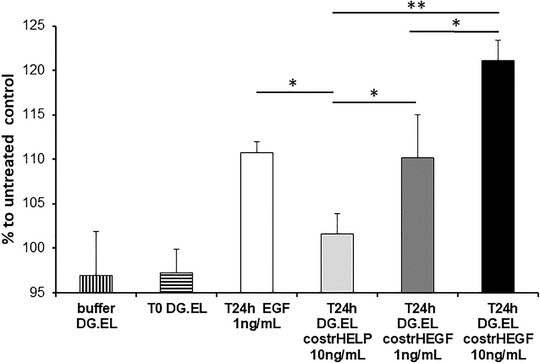
Assessment of EGF activity that was released from HEGF‐loaded alginate (AL) composites on HaCaT cell proliferation. Cultures were treated with the digestion buffer (DG) without elastase and with the digestion buffer with elastase in which the HEGF‐containing AL composites were incubated for 24 h (T24h DG.EL costrHEGF). As controls, cells were treated with buffer alone (bufferDG.EL), and with the supernatant collected at the beginning of the incubation with elastase (T0 DG.EL). For additional control, cells were treated with the supernatant from the composite embedded in HELP matrix without HEGF (T24h DG.EL costrHELP) as well as with a commercially available EGF (T24h EGF). Results are expressed as mean ± standard deviation; the analyses were performed at least in quadruplicate. *p < 0.05; **p < 0.01

Following the same procedure used to assess the HEGF bioactivity (Figure 2), the HaCaT cells were treated with the supernatants from digestion with elastase in which the HELP/HEGF matrix‐embedded constructs were incubated. The result of the analysis is shown in Figure 8. The supernatants containing EGF, which was roughly estimated by the dot blot procedure (see Materials and Methods for details), showed a proliferative effect comparable to that of EGF on the cells. As already observed, the digestion supernatants from the composite embedded in HELP alone showed only a slight effect, confirming a minor nonspecific effect of the elastin‐like polypeptide on cell proliferation.^26^, ^34^

This study showed that these new polysaccharide‐based composites possess not only stimuli‐responsive properties but also maintain bioactivity. They appear particularly suitable for the controlled release of molecules carrying peptidic domains endowed with biological activity. These composites represent promising delivery platforms that allow exploiting the interaction with disease‐associated signals, like those that arise during inflammation. This condition is characterized by the presence of elastolytic activity that can trigger and activate the targeted release. This system offers several advantages over conventional delivery routes, since the bioactive domain is stored within the matrix, the nonspecific distribution of the therapeutic principle is reduced as well as the dose and the frequency of administration. Notably, the observation that the two described AL scaffold composites showed two distinct bioactive domain release kinetics suggests that further control of the release is achievable. The fine tuning of the scaffold structure by 3D printing is likely to result in further control of the release of the bioactive principle.

## 4 CONCLUSIONS

The supplementation of therapeutic adjuncts like the growth factors has gained rising attention in contexts such as those of chronic and hard‐to‐heal skin lesions. To explore alternative routes of administration of bioactive factors that ensure appropriate and continued supply of the effective principle at the local level, we designed new bioactive composites based on polysaccharide scaffolds that respond to the presence of proteolytic stimuli releasing the functional EGF domain.

The preparation and characterization of composites based on 3D‐printed polysaccharide scaffolds and HELP stimuli‐responsive biopolymers are described. The procedure to obtain these composites involved multiple steps that did not affect the functionality of the bioactive domain that was integrated within the constructs. After the degradation triggered by the proteolytic stimuli that were present in the environment, the functional EGF domain was released from the composites and the biological activity was assessed.

Notably, this approach has great flexibility, in that 3D printing allows to further modulate the structure at the micro/nanoscale as well as to employ different materials. On the other hand, the HELP system, beyond enabling the direct integration of any bioactive region of interest, allows the mixing of more than one bioactive HELP‐fusion protein without the loss of matrix‐forming and stimuli‐responsive properties.

Altogether, the features of the described composites are particularly appealing for the topical delivery of adjuncts at the level of hard‐to‐heal skin lesions, overcoming drawbacks like high and repeated dose administration and reducing the likelihood of severe side effects. Ultimately, our system represents not only a novel setup but also a model that can be applied to many different cutaneous refractory conditions, like the bacterial and fungal infections and autoimmune and inflammatory skin lesions.

## CONFLICT OF INTEREST

The authors declare that there is no conflict of interest.
